# Are Nurses Necessary?

**Published:** 1920-11-13

**Authors:** 


### ARE NURSES NECESSARY?

To borrow a war expression, there is a certain liveliness in the hospital world. Slowly but surely the fact that something serious is wrong is being borne in on the public mind. One hears and sees comments in all sorts of unexpected places. The Minister of Health last week in Parliament gave startling figures showing the excess of expenditure over income, and Lord Knutsford's staccato announcement that the "London" will close on New Year's Day will cause many ears to tingle.

It is high time that there was an awakening, and let it not be forgotten that the great majority are still sleeping. It is worthy of note that although the nurse is one of the chief features of the hospital problem her name has not yet been mentioned. Hence, despite the fact that even in Editorial G.H.Q. there is a tendency to regard a State Nursing Service as a Utopian dream, rather than a businesslike and practical suggestion, I am disposed to the opinion that the suggestion is seasonable. The attitude and action of nurses at the present acute crisis are matters of the utmost importance. Their case is not likely to receive serious consideration unless they present it themselves.

The nurses' case is twofold in character. It is both subjective and objective. It concerns the members of the profession as individuals, but it also concerns the general public, and much more acutely. If a nurse is turned adrift she is almost sure to find some other employment. The sick and suffering public will find it extremely difficult to obtain a suitable substitute for a trained nurse. And what this crisis means is that the trained nurse may have to pack her bag and disappear.

### The Bread-and-Butter Argument.

I pray the reader not to regard this as exaggerated language. What effect, think you, is Lord Knutsford's announcement—be it something actual, or only a threat—going to produce among candidate probationers? What will be the effect of the thousand and one S.O.S. cries from voluntary hospitals? After all, the bread-and-butter argument counts for much, and young women of energy and capacity will hesitate before embarking on a nursing career while the waves are mountains high, and the captains are threatening to abandon the ships. Those who have the interests of the profession at heart ought to devote their energies either to finding a remedy while yet there is time, or else to providing rafts for the shipwrecked.

### Pity the Ratepayer!

In offering objections to the Ministry of Health Bill last week, Lord Hugh Cecil made a remark which sets out with clearness and brevity a sound doctrine which no reformer can afford to ignore. "It is a profound error," he said, "to think that it is now the time to spend money on things which are more or less desirable. Bills should contain only that which is absolutely indispensable for the exigencies of the moment. Before bringing in Bills to do certain things they (the promoters) must prove that the things are so urgently necessary that great evils would ensue if they were not done." This is the text on which the claim for a State Nursing Service must be based.

### Dreamland or Utopia.

If the conditions named are absent then dreamland or Utopia is the name of the country to which it may apply. But if they are not absent it is a waste of sentiment to pity the ratepayer. Nurses must live. But it is not in order that nurses must live that a State service is advocated. It is in order that society may be reconstructed on a strong foundation of physical health. The ratepayer has consented to an annual police bill of seventy millions. He has insisted on a first-class scheme of education, at an increased cost, according to *The Times*, of 200 per cent. He is paying 240 per cent. more for his railways and 215 per cent. more for the maintenance of the armed forces of the Crown than in pre-war days.

### Destruction or Reconstruction?

Is the nurse to be scrapped because of these things? If she is, it means that the nation is suffering from obliquity of vision. It is obviously absurd to pay on an elaborate scale for everything in the world save health, and to relegate this to a position even more obscure than it occupied before the war. Badly off as hospitals may have been, nobody talked of closing them until we entered on the gigantic enterprise of Reconstruction!

One grows accustomed to shocks. It was certainly a shock to me when I realised that the Ministry of Health preferred college women to trained nurses as health visitors. And the nurse took it lying down! She has gained nothing by the passing of the Sex Disqualification (Removal) Act, and suffers because there is no Nurse Disqualification (Removal) Act.

It seems to me that the question ought to be put to the people of this country: Is nursing a national necessity for the sick, or shall we dispense with nurses and revert to the system described, I think, in "Lorna Doone," by which the survival of the fittest was insured by throwing the weaklings over the battlements?

IERNE.

